# Visual, Auditory, and Cross Modal Sensory Processing in Adults with Autism: An EEG Power and BOLD fMRI Investigation

**DOI:** 10.3389/fnhum.2016.00167

**Published:** 2016-04-19

**Authors:** Elizabeth’ C. Hames, Brandi Murphy, Ravi Rajmohan, Ronald C. Anderson, Mary Baker, Stephen Zupancic, Michael O’Boyle, David Richman

**Affiliations:** ^1^Department of Electrical and Computer Engineering, Texas Tech University, LubbockTX, USA; ^2^Department of Audiology, Texas Tech University Health Sciences Center, LubbockTX, USA; ^3^Department of Pharmacology and Neuroscience, Texas Tech University Health Sciences Center, LubbockTX, USA; ^4^College of Human Sciences, Texas Tech University, LubbockTX, USA; ^5^Burkhart Center for Autism Education and Research, Texas Tech University, LubbockTX, USA

**Keywords:** autism, EEG, fMRI, cross-modal sensory processing, visual, auditory

## Abstract

Electroencephalography (EEG) and blood oxygen level dependent functional magnetic resonance imagining (BOLD fMRI) assessed the neurocorrelates of sensory processing of visual and auditory stimuli in 11 adults with autism (ASD) and 10 neurotypical (NT) controls between the ages of 20–28. We hypothesized that ASD performance on combined audiovisual trials would be less accurate with observable decreased EEG power across frontal, temporal, and occipital channels and decreased BOLD fMRI activity in these same regions; reflecting deficits in key sensory processing areas. Analysis focused on EEG power, BOLD fMRI, and accuracy. Lower EEG beta power and lower left auditory cortex fMRI activity were seen in ASD compared to NT when they were presented with auditory stimuli as demonstrated by contrasting the activity from the second presentation of an auditory stimulus in an all auditory block vs. the second presentation of a visual stimulus in an all visual block (AA2-VV2).We conclude that in ASD, combined audiovisual processing is more similar than unimodal processing to NTs.

## Introduction

Autism spectrum disorder (ASD) represents a potentially life long condition that is defined by the American Psychiatric Association as containing three key features: (1) restricted interests (2) repetitive behaviors and (3) impaired social communication (American Psychiatric Association, 2013). Arguably, deficits in social communication pose the greatest challenge for affected individuals in their attempts to maintain healthy and productive social interactions. Therefore, attempts to identify a neurological basis for these deficits have received a great deal of investigation in recent years (see Amaral et al., 2008; Anagnostou and Taylor, 2011; Kana et al., 2011 for review). Amongst these investigations, a common trend appears to involve dysfunctions in sensory processing (Iarocci and McDonald, 2006). For a comprehensive review of how sensory processing is affected in individuals with ASD, please see Marco et al. (2011). In brief, sensory processing dysfunctions have been noted in ASD since the original reports by Kanner (1943) and Asperger (1944). Difficulties in sensory behavioral responses, much like other symptoms of the spectrum disorder, range from mild to severe, and may persist throughout adulthood (Minshew et al., 2002; Blakemore et al., 2006; Leekam et al., 2007; Tomchek and Dunn, 2007; Crane et al., 2009). These difficulties may lead to self-injurious or aggressive behavior when individuals with ASD become frustrated by their inability to communicate their experiences. Although, there does not appear to be a consistent pattern of sensory deficits in ASD, deficits are more prevalent in these individuals than in those that are affected by other developmental disabilities (Baranek et al., 2006; Leekam et al., 2007; Ben-Sasson et al., 2009).

Evaluation of sensory processing and the integration of sensory processing across visual and auditory modalities can be compared in several ways when assessing differences in individuals with ASD and neurotypical (NT) controls. One key issue involves whether or not the auditory and/or visual information was presented in a social context. Another issue involves the mode of presentation of the stimuli: whether they are auditory, visual, or mixed. Responses of ASD individuals to auditory and/or visual, stimuli both within and outside of social context are summarized in the following sections.

Given that difficulties with language and non-verbal social cues (e.g., body language and facial expressions) are often a source of problems for individuals with ASD in social situations, investigations of auditory and visual processing have received a great deal of attention. One common method of observing auditory processing is by measuring the auditory brainstem response through the use of surface electrodes. These electrodes record electrical activity generated by the neurons of the brainstem in response to hearing a series of clicks and tones. While reports of latency and amplitude vary (Rosenhall et al., 2003; Kwon et al., 2007; Dunn et al., 2008), Källstrand et al. (2010) saw that adults with ASD had a right-sided asymmetrical attenuation of wave III amplitude that separated these individuals from control, schizophrenic, and attention deficit hyperactivity disorder individuals; suggesting abnormal lateralization preferences in affective auditory pathways via the subcortical and brainstem nuclei that could lead to aberrant perception of auditory stimuli. Furthermore, children with ASD have been shown to have typical brainstem responses to clicks, but differential responses to varied pitch and speech sounds (Russo et al., 2008, 2009). Together, these studies suggest that while basic auditory processing by the brainstem may explain some of the abnormalities in language experienced by individuals with ASD, much of the difficulty may be attributable to dysfunctions in higher level cerebellar and cerebral cortical processes.

Similar to investigations of auditory processing, there are conflicting results from visual processing studies. While some studies suggest there is no difference in the processing of low or high spatial frequencies with regard to the motion or form of objects in individuals with ASD compared to NTs (De Jonge et al., 2007; Koh et al., 2010), other studies indicate that individuals with ASD have impairments in object boundary detection (Vandenbroucke et al., 2008), contrast detection (Sanchez-Marin and Padilla-Medina, 2008), and undifferentiated responses for mid vs. high-level spatial frequency gratings (Jemel et al., 2010), as indicated by evoked visual potentials. Much like the observations on auditory processing, these findings suggest that an inability to successfully integrate incoming stimuli becomes an increasing challenge in more complicated and nuanced tasks.

Also similar to the observations made in auditory processing, differences are noted in the way individuals with ASD process visual stimuli related to human interaction compared to inanimate objects. For example, children with autism were shown to have impairments in the processing of dynamic noise, motion coherence, and form-from-motion detection by Annaz et al. (2010). Further support comes from work by Parron et al. (2008), which observed that children with ASD differed only from neurotypical children in their ability to name emotional point-light displays. Similar to the findings of auditory investigations, such evidence supports the notion that dysfunctions in basic sensory processing likely exist, but are more susceptible to emotionally suggestive stimuli (e.g., changes in voice pitch or facial expressions) than audio or visual presentation of non-human objects.

Lower level multimodal processing refers to the brain’s ability to successfully integrate stimuli from different modes of presentation (e.g., touch vs. sight) into a single coherent experience (e.g., biting into an apple). A previous investigation by van der Smagt et al. (2007) used a “flash-beep” paradigm, in which individuals with ASD were believed to have exhibited the same illusion phenomenon experienced by NTs. During this procedure, multiple auditory tones are paired with a single transient visual flash. This leads to an illusion that multiple flashes are present; thus representing an error in cross modal processing that is common in NTs. Observing the same behavioral outcome in individuals with ASD led to the conclusion that cross modal processing at this level is essentially similar. Had this been true, it would further cement the notion that difficulties in sensory processing for these individuals are restricted to more socially dependent interpretations of stimuli. However, Foss-Feig et al. (2010) later demonstrated that individuals with ASD continued to experience this illusion even when the length of the time interval between the auditory tones and visual flash were sufficient to negate this experience in NTs. These findings were congruent with an electroencephalography (EEG) study conducted by Courchesne et al. (1985) that reported a reduction in response amplitude (compared with typically developing children) during simultaneous presentation of auditory and visual stimuli. This study found that multimodal sensory processing in children with ASD tended to deviate from NT children at the later stages of sensory integration where stimuli are collapsed to allow for more efficient processing and highlighted that such deficits were not limited to socially cued stimuli.

Higher level multimodal processing differs from lower-level multimodal processing in that it incorporates cognitive processes such as logical reasoning or recognition of emotional cues in addition to the integration of multiple types of sensory processing to understand an event as it takes place (e.g., Inferring what someone has said through the context of a conversation even when some words cannot be heard). Deficits in higher-level multimodal processing are therefore believed to be more reliant upon socially cued inferences and may be responsible for the difficulties in speech production and comprehension often observed in this population (Marco et al., 2011). Individuals with ASD rely less on the lip reading portion of the McGurk effect and therefore fail to show improvement when trained on the visual feedback component (Smith and Bennetto, 2007; Iarocci et al., 2010). Although it may not be consciously acknowledged, reliance on lip reading is a common mechanism used by NTs when attempting to comprehend instructions in a noisy or distracting environment. In fact, Mongillo et al. (2008) showed that individuals with ASD were capable of processing audio and visual information related to bouncing balls just as well as NTs, but were not as successful when it came to human facial expressions and voices, further solidifying the disconnect in higher-level processing of socially mediated stimuli. Investigations of a neuroanatomical basis for multimodal sensory processing deficits have identified several likely cortical regions including the prefrontal cortex and temporal association cortices (Foxe and Molholm, 2009) making them likely structures of interest. Neuroimaging techniques, therefore, represent a powerful tool that may resolve these questions regarding the structural or functional basis of multimodal processing impairment in ASD.

An example of how neuroimaging has contributed to our understanding of ASD may be seen in investigations involving face processing, as it represents one of the best studied visual aspects of social interaction in individuals with autism (Schultz, 2005). Two studies by Dalton et al. (2007) used fMRI and eye tracking technology to show reduced activations in the fusiform gyrus and the amygdala in individuals with ASD and their siblings and that the amount of activation in these regions positively correlated with fixation time on the eye region of the face (Dalton et al., 2005).

The goal of the current study was to determine the neural correlates of cross modal audiovisual processing in young adults (ages 20–28) with ASD as measured by behavioral accuracy, EEG power, and blood oxygen level dependent functional magnetic resonance imagining (BOLD fMRI) using simple auditory tones and visual shapes. EEG power was chosen over evoked potential as the investigative method used in this study because it allows for a more direct observation of how the brain processes information instead of how the brain receives different types of information in different ways.

The use of simple auditory and visual stimuli allowed us to determine differences between individuals with ASD and NT controls in processing auditory, visual, and mixed auditory–visual information by using a task that did not incorporate complex socially charged stimuli. This approach allowed us to specifically isolate visual and auditory processing and visual/auditory sensory integration processes. Based on the literature presented, we hypothesized that while adults with ASD would likely show reduced EEG power for the alpha and beta bands and reduced BOLD fMRI activations in the occipital and temporal regions for unimodal presentation of visual and auditory stimuli respectively, the greatest reductions in EEG power and BOLD activation compared to NTs would be observed within the frontotemporal regions during simultaneous presentation of stimuli, highlighting their uniquely plausible role in the integration of multimodal stimuli.

## Materials and Methods

### Participants

Ten NT individuals and eleven individuals with ASD between the ages of 20 and 28 participated in both the EEG and fMRI study sessions. Three subjects from the ASD group and eight from the control group were women. One subject from the ASD and one from the control group were ambidextrous: FA13025, FC12002. One subject from the ASD group was left-handed: SA13022. One subject from the ASD group was diagnosed via ADOS by a local licensed psychologist; the remaining subjects were diagnosed through various means and screened by the transitional living facility/schools through testing, but we were not privy to the results. However, individuals identified through the transitional living program had been approved by the state of Texas to receive services based on an autism diagnosis. All participants passed a hearing screening to ensure audibility of the tones presented during the study. The hearing screening was employed with an ANSI (American National Standards Institute) calibrated screener for a range of 250 Hz through 8,000 Hz. All participants completed a handedness questionnaire (Oldfield, 1971). This study was approved by the Human Subjects Internal Review Board at Texas Tech University with written informed consent from all subjects in accordance with the Declaration of Helsinki.

### Sensory Task

**FIGURE 1:**
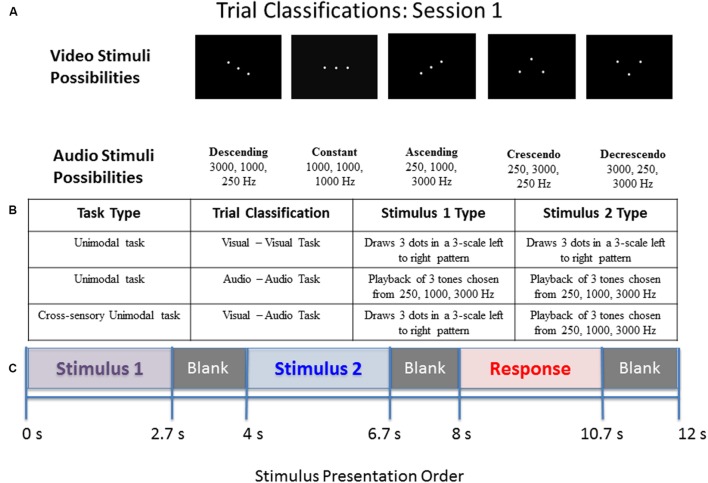
**Diagram of stimulus presentation design for session 1.** **(A)** Depiction of the five pattern types of visual dots (top) or auditory tones (bottom) that participants saw/heard: Descending, Constant, Ascending, Crescendo, or Decrescendo (from left to right). **(B)** Description of the possible trial conditions presented in session 1. **(C)** Schematic of study design. During the “response” segment the participant pressed a button to indicate whether the tones or dots in pattern 1 were the same as those in pattern 2.

**FIGURE 2:**
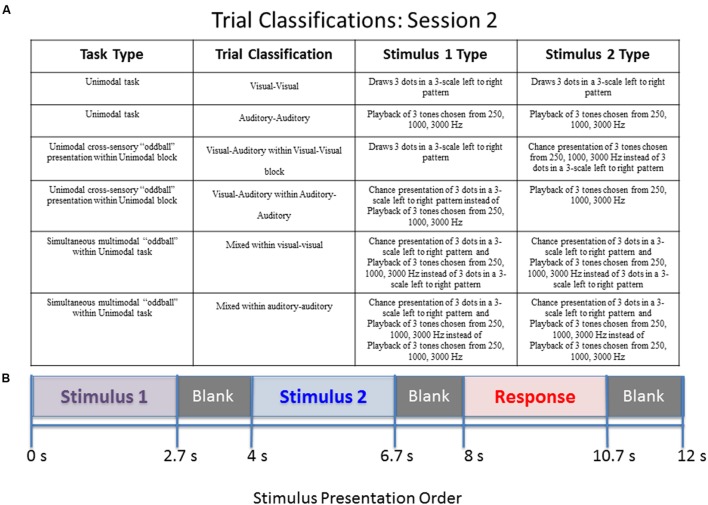
**Diagram of stimulus presentation design for session 2.** **(A)** Description of the possible trial conditions presented in session 2. Mixed within auditory–auditory (oddball visual) – an auditory tone sequence followed by an auditory sequence 70% of the time, and a simultaneous audio–visual oddball 30% of the time. Mixed within visual–visual (oddball auditory) – a visual pattern followed by a visual sequence 70% of the time, and a simultaneous audio–visual oddball 30% of the time. **(B)** Schematic of study design. During the “response” segment the participant pressed a button to indicate whether the tones or dots in pattern 1 were the same as those in pattern 2.

Across the different trial conditions, participants were presented with a visual stimulus in the form of three dots on a screen (in relative positions of low, middle, high), an auditory stimulus in the form of three pure tones (250, 1000, or 3000 Hz), or both. Each stimulus presentation, whether auditory, visual, or both, consisted of one of five pattern types: descending, constant, ascending, crescendo, or decrescendo. Participants were asked to determine if the pattern displayed from the first stimulus presentation matched the pattern displayed by the second via a button press. Whenever both dots and tones were presented simultaneously, they were of the same pattern type (as this represented a single presentation of a multimodal stimulus). For all blocks of both sessions, the first stimulus was presented for 2.7 s, followed by an inter-stimulus interval (ISI) of 1.3 s, the second stimulus for 2.7 s, a second ISI for 1.3 s, a response screen for 2.7 s, and finally an ISI for 1.3 s. The study paradigms for sessions 1 and 2 are illustrated in **Figures 1** and **2**, respectively.

Therefore, the sensory task was designed to be completed in two consecutive sessions. Both sessions were counterbalanced. The first session lasted approximately 10 min. The frequencies of the pure tones were selected to avoid distortion and masking effects of the fMRI mechanical noise (250, 1000, and 3000 Hz). The frequencies and intensity allowed for compensation of the attenuation provided by the hearing protection required for all fMRI participants.

Stimuli within session 1:

(1) Visual–Visual Stimulus (VV): patterns 1 and 2 are both three dots occurring in sequence.
(2) Auditory–Auditory Stimulus (AA): patterns 1 and 2 are both three pure tones played in sequence (with a blank screen).
(3) Visual–Auditory Stimulus (VA): pattern 1 is three dots occurring in sequence and pattern 2 is three pure tones played in sequence (with a blank screen).

The first session consists of three blocks of stimuli. The first block contains 20 visual–visual stimuli, the second block contains 20 auditory–auditory stimuli, and the third block contains 20 visual–auditory stimuli. The main goal of session 1 was to determine what happens when individuals have to switch sensory modalities (from visual to auditory) when making a decision about pattern matching, and whether those with autism perform differently when crossing from visual to auditory processing.

The second session also contained some unimodal stimulus presentations for internal consistency, but more importantly contained trials of mixed (simultaneous) audio/visual stimulus presentation. This session lasted approximately 7 min. The order of events during a stimulus is identical to the first session given in **Figure 1**. Four types of possible pattern matching are presented in the second session, given in the following list.

Stimuli within session 2:

(1) Visual–Visual Stimulus (VV): patterns 1 and 2 are both three dots occurring in sequence.
(2) Auditory–Auditory Stimulus (AA): patterns 1 and 2 are both three pure tones played in sequence (with a blank screen).
(3) Visual–Auditory Stimulus (VA): pattern 1 is three dots occurring in sequence and pattern 2 is three pure tones played in sequence (with a blank screen).
(4) Mixed Stimulus (MM): pattern 1 is three dots occurring in sequence and three pure tones played simultaneously. The simultaneous dots and tones have matching patterns. Pattern 2 is also three simultaneous dots and tones.

The second session consists of two blocks of stimuli. The first block contains 14 visual–visual stimuli, 3 visual–auditory stimuli, and 3 mixed stimuli. The three types of stimuli are randomly mixed into the block. The second block contains 14 auditory–auditory stimuli, 3 visual–auditory stimuli, and 3 mixed stimuli, also all randomly mixed into the block. The visual–auditory and mixed stimuli are meant to be oddball events. The task was designed to assess what happens when individuals have to switch sensory modalities in a randomized manner, and also to assess the impact of simultaneous visual and auditory information. A key to the abbreviations for each of the trial types is provided in **Table 1**. Further explanation of the session 2 paradigm is provided in **Figure 2**.

**Table 1:** Event abbreviations for stimulus presentations.

Session 1			Session 2		
	
Stimulus	Pattern 1	Pattern 2	Stimulus	Pattern 1	Pattern 2
Visual–Visual	VV1	VV2	Visual–Visual	VV1	VV2
Auditory–Auditory	AA1	AA2	Auditory–Auditory	AA1	AA2
Visual–Auditory	VA1	VA2	Visual–Auditory within Visual–Visual block	VV_VA1	VV_VA2
			Visual–Auditory within Auditory–Auditory	AA_VA1	AA_VA2
			Mixed within Visual–Visual	VV_MM1	VV_MM2
			Mixed within Auditory–Auditory	AA_MM1	AA_MM2


The same sensory task was used for both the EEG and fMRI studies and was presented through E-Prime 2.0. EEG was performed first. fMRI was performed within a day; if not on the same day. Participants were allowed to practice using the clicker to respond to the cues with an example trial, but were not explicitly trained before test sessions. The responses were monitored for accuracy. In order to properly assess differences in multimodal processing between the two groups it was necessary to observe unimodal sensory processing [as represented by the auditory–auditory (AA) and visual–visual (VV) trials] and cross sensory processing of unimodal stimulus presentation [represented by visual–auditory (VA) trials]. To clarify, cross sensory processing of unimodal stimulus presentation means that only one mode of stimulus (e.g., visual or auditory) is presented at a given time (i.e., unimodal), but is then followed by a unimodal presentation of the alternative type of stimulus (e.g., auditory followed by visual or visual followed by auditory).

## Behavioral Analysis

### EEG Session

Participants’ scores were averaged for each trial type within sessions 1 and 2. One NT participant’s responses were lost due to a corrupted data file. The scores represent whether the answer given by the participant as to whether or not the patterns matched were correct. Scores were separated for same and different patterns. A two-way ANOVA was used to identify stimuli where the NT group had significantly different scores than the ASD group for a False Discovery Rate corrected, *p* < 0.05.

### BOLD fMRI Session

The same method of analysis used for EEG stimulus scores was implemented on the fMRI stimulus scores. Participant responses from all participants were recorded and analyzed.

### EEG Methodology

#### EEG Procedure

Participants completed the sensory task while undergoing EEG recording. The EEG was acquired with a 65-channel EGI HydroCel Geodesic Sensor Net with a sampling rate of 500. Stimuli were presented on a 10.25″ by 13.25″ monitor. The task audio stimuli were presented through a set of ANSI regulated insert earphones. Participants were given a four-button keypad to record their responses. They were asked to only use buttons one and two to record their “same” or “different” decision. The presentation software tracked scores and response times as participants responded.

#### EEG Power Pre-processing

Electroencephalography data were filtered using a high-pass IIR filter at 1 Hz and a low-pass IIR filter at 50 Hz using standard open source software, EEGLAB. The data were then visually inspected to remove bad portions of data with large numbers of artifacts like eye movements or noise. A method known as independent component analysis (ICA) was used to help identify and remove artifactual components from the data. This was done using EEGLAB’s *runica* algorithm.

#### EEG Power Processing

After artifact removal, the data were divided into events. Each event consisted of patterns 1 and 2 for each of the unique stimuli within sessions 1 and 2 of the sensory task. **Tables 2** and **3** give the types of events within each session and their abbreviations used throughout the results section. Within the events, data were further sub-divided into 1 s segments referred to as epochs. One-second epochs were created using the 1st second, 2nd second, and 3rd second of each event to capture variations in response as measured by EEG power over time.

**Table 2:** Session 1 alpha EEG power event differences by group.

Comparison	Control		Autistic	
		
	Higher power	Region (channel no.-area)	Higher power	Region (channel no.-area)
AA1-VV1	AA1	All		
AA1-VA1	AA1	All		
AA2-VV2	AA2	All		
VA2-VV2	VA2	All		
VV1-VA1			VA1	3-LT,14-RFP
AA2-VA2	VA2	6-RPA, 9-LC, 10-RC, 11-LF, 13-LFP		


*3-LT, Left temporal; 6-RPA, right posterior auricular; 9-LC, left central; 10-RC, right central; 11-LF, left frontal; 13-LFP, left frontal pole; 14-RFP, right frontal pole.*

**Table 3:** Session 1 beta EEG power event differences by group.

Comparison	Control		Autistic	
		
	Higher power	Region (channel no.-area)	Higher power	Region (channel no.-area)
AA1-VV1	AA1	All	AA1	5-LPA, 9-LC
AA1-VA1	AA1	1-FC, 2-PC, 3-LT, 4-RT, 5-LPA, 6-RPA, 7-LO, 8-RO		
AA2-VV2	AA2	All	AA2	5-LPA
VA2-VV2	VA2	All	VA2	3-LT
VV1-VA1	VA1	3-LT	VA1	All
AA2-VA2	VA2	1-FC, 3-LT, 6-RPA, 9-LC, 10-RC, 11-LF, 13-LFP, 14-RFP	VA2	3-LT


*1-FC, Frontal central; 2-PC, posterior central; 3-LT, left temporal; 4-RT, right temporal; 5-LPA, left posterior auricular; 6-RPA, right posterior auricular; 7-LO, left occipital; 8-RO, right occipital; 9-LC, left central; 10-RC, right central; 11-LF, left frontal; 13-LFP, left frontal pole; 14-RFP, right frontal pole.*

**FIGURE 3:**
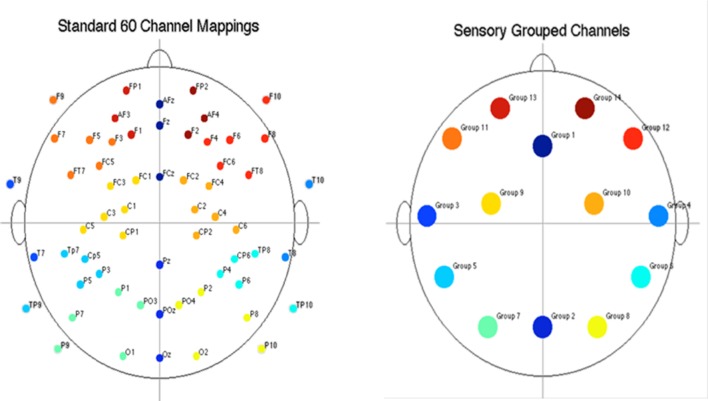
**Diagram of electrode placement and channel grouping for data reduction purposes.** Original 60 channels (top) and reduction to 14 channel groups (bottom).

Electroencephalography RMS (root-mean-square) power was computed for each participant, for each EEG channel, and for each epoch within the alpha (8–12.6 Hz) and beta (13–30 Hz) frequency bands. The power computations were averaged within events for each participant. To reduce the amount of data, EEG channels were grouped into fourteen regions, shown in **Figure 3**. Participants’ channel power was averaged for each group of channels. After grouping channels, each participant had 2,268 power measures (14 channel regions × 3 frequency bands × 18 events × 3 epochs). Given that the presentation and processing of the stimuli would take approximately 1 s, the results presented in this section are computed using the “2nd second” epochs (i.e., 1 s after stimuli onset), with EEG power levels averaged across similar events, resulting in 14 power measures per frequency band per event type.

#### EEG Power Post-processing

Three types of statistical analysis were performed. First, group main effects for sessions 1 and 2 were tested using repeated measures ANOVA (rANOVA) where group (autistic or control) was the independent variable, EEG power was the dependent variable, and event type was the within-subject factor. The rANOVA was first implemented without respect to channel region (as a global measure). Next, it was implemented for each channel region separately. Along with group main effect, the interaction between group and event was tested for sessions 1 and 2. Second, *post hoc* testing was performed to determine significant differences between group means on each type of event within sessions 1 and 2. This was also performed with and without distinction of channel region. Significant differences were determined using the Tukey–Kramer method for *p* < 0.05. Third, *post hoc* testing was also performed to determine significant differences between events within groups (i.e., autistic AA1 vs. autistic VA1 or control AA1 vs. control VA1) for each channel region. Significant differences were determined using the Tukey–Kramer method for *p* < 0.05.

### BOLD fMRI Methodology

#### BOLD fMRI Procedure

The task visual stimuli were projected onto a screen above each participant. The auditory stimuli were presented through headphone speakers allowing the intensity to compensate for the attenuation provided by the hearing protection and to avoid masking effects from the mechanical noise. Participants were given a two-button keypad to record their responses. The presentation software tracked scores and response times as participants responded.

#### Image Acquisitioning

In a separate session, participants underwent BOLD fMRI. A structural image was obtained first, and then the same auditory–visual task described above was administered to obtain functional (BOLD fMRI) data. The settings on the three Tesla Skyra MRI system were as follows:

T1-weighted structural parameters: TR = 1900 ms, TE = 2.49 ms, flip angle = 9°, FOV = 240, slices = 192, and voxel size = 0.9 mm × 0.9 mm × 0.9 mm.

T2^∗^ Echo-planar Imaging (EPI) parameters: TR = 2000 ms, TE = 20 ms, flip angle = 80°, FOV = 240, slices = 41, and voxel size = 3 mm × 3 mm × 3.5 mm. fMRI images were acquired in an ascending fashion (inferior to superior) without a gap between slices.

#### Image Pre-processing

Functional data analyses were carried out using Statistical Parametric Mapping 8 (SPM 8; Wellcome Department of Cognitive Neurology, London UK, http://www.fil.ion.ucl.ac.uk/) in MATLAB. For both sessions of the sensory study, each participant’s first functional image was corrected for the anterior commissure (AC) coordinates. All remaining images in the sequence were realigned to the first image. Participants’ structural images were also corrected for AC coordinates. The mean functional images were co-registered to the reoriented structural images, segmented using the SPM8 default tissue probability maps, and registered and normalized to standard Montreal Neuroimaging Institute (MNI) space. Finally, functional images were smoothed using a 10 mm FWHM Gaussian kernel filter.

#### Image Processing and Post-processing Statistical Analyses

After preprocessing, fMRI images were analyzed using an event-related model with a general linear model assumption. The events in sessions 1 and 2 were modeled as regressors in the general linear model. Contrast images were generated at the first level (individually) for the following main effects:

Session 1 contrasts:

(1) VV1-VA1
(2) VV2-VA2
(3) AA1-VA1
(4) AA2-VA2
(5) VV1-AA1
(6) VV2-AA2

Session 2 contrasts:

(1) VV1-VV_VA1
(2) VV2-VV_VA2
(3) AA1-AA_VA1
(4) AA2-AA_VA2
(5) VV1-VV_MM1
(6) VV2-VV_MM2
(7) AA1-AA_MM1
(8) AA2-AA_MM2

Second level contrast images were generated for within and across group effects (ASD vs. NT) using one-sample and two-sample *t*-tests on the first level contrast images. Results were based on a family wise error (FWE) corrected *p*-value threshold of *p* < 0.05 and k = 5 voxel threshold. Differences between control and autistic group activations occurred when comparing stimulus events across modes. Activation coordinates were then transformed into Brodmann areas (BA).

## Results

### Behavioral Results

#### EEG Sessions

**Supplementary Figure S1** shows the average accuracy scores for the NT and ASD groups for the EEG sessions. No significant differences were observed between the NT and ASD groups across any trial types or sessions. The general trend shows that the ASD group had lower average scores than the NT group, with a much higher standard deviation, and that the ASD group had lower average scores for when the patterns were the “same” stimuli than when the stimuli were “different” (i.e., SVV vs. DVV; SAA vs. DAA).

#### fMRI Sessions

**Supplementary Figure S2** (Top) shows the average scores for the NT and ASD groups for sessions 1 and **Supplementary Figure S2** (Bottom) shows the average scores for session 2. No trial condition resulted in significantly different scores between the NT and ASD groups in either session.

### EEG Power

#### Single Stimulus Presentation Trials

**FIGURE 4:**
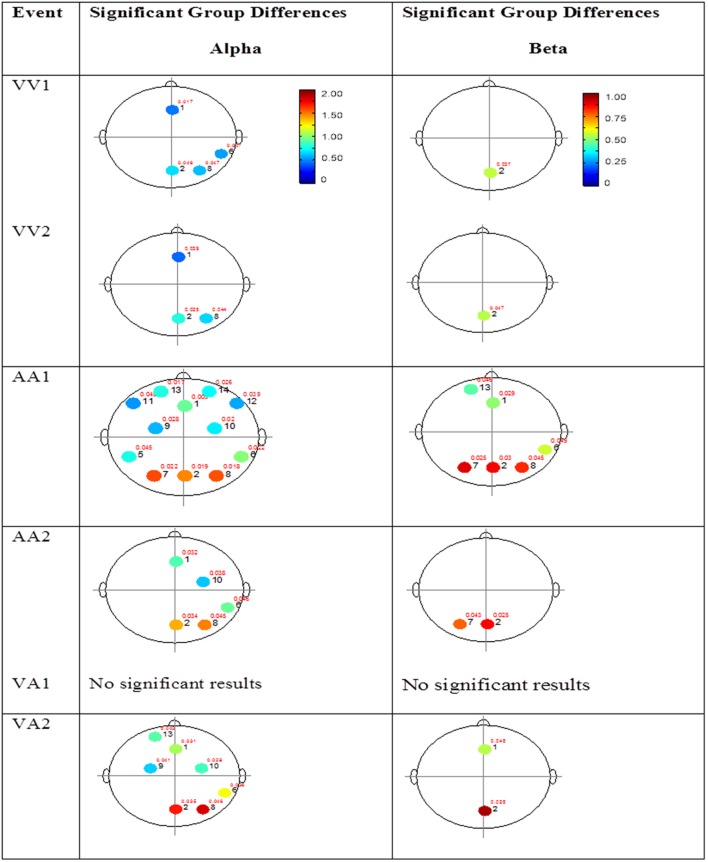
**Session 1 alpha and beta EEG power group differences.** Results for the alpha band **(left)** and beta band **(right)** are presented. Significant differences were determined using the Tukey–Kramer method for *p* < 0.05.

The results for both the alpha and beta bands are summarized in **Tables 2** and **3**, respectively. For additional assistance in visualizing channel locations presented in tables, please refer to **Figure 3**. EEG average power head plots are presented in **Figure 4**. Across all channels for all events, EEG average power was lower for the ASD group (graphs of average EEG power). Solely visual tasks displayed little, but consistent, difference between the ASD and NT groups across both bands. For the alpha band frequency, channel groups 1 (medial frontal), 2 (medial occipital), and 8 (right temporal) were shown to be less active in the ASD group. For the beta band frequency, channel group 2 was slightly less active for the ASD group. Lastly, the “first” vs. “second” (VV1 vs. VV2) stimulus presentation generated identical maps of average power, suggesting presentation order made no difference in how these stimuli were processed by the ASD group. On the other hand, tasks containing an auditory stimulus were consistently observed to display significant differences in average power across multiple channel groupings for both the alpha and beta bands. For the alpha band frequency, the ASD group was shown to have decreased activity across all regions: frontal (1, 11, 12, 13, and 14), parietal (9 and 10), temporal (5 and 6), and with the greatest differences observed for the occipital (2, 7, and 8) channels during the “first” (AA1) stimulus presentation. A similar, but attenuated, pattern was seen for the “second” (AA2) stimulus presentation. Lastly, the “second” cross modal stimulus presentation (VA2; i.e., the auditory portion of the cross modal block) produced a similar, seemingly intermediary, pattern to AA1 and AA2. Recordings of beta band frequencies produced similar, yet less extensive, patterns of deactivation for each of the corresponding audio containing trials.

#### Mixed (Simultaneous) Presentation Trials

**FIGURE 5:**
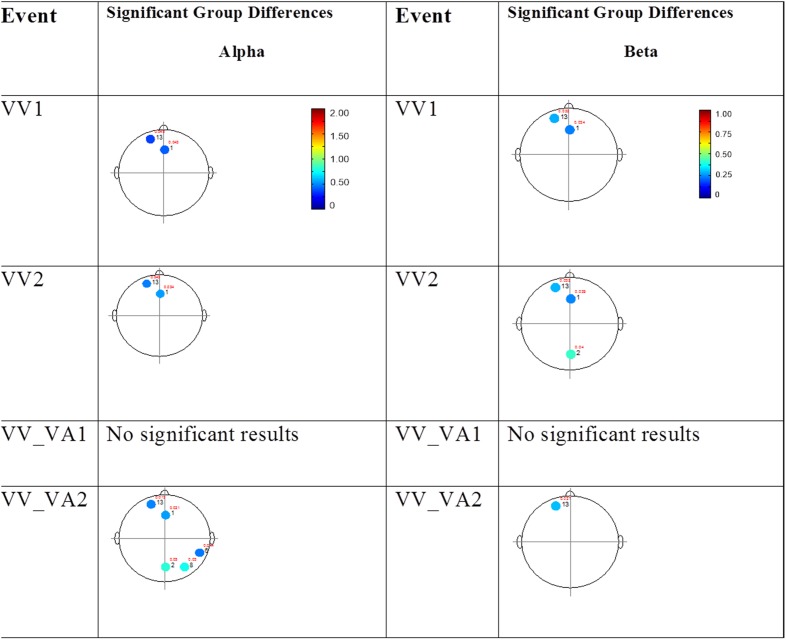
**Session 2 alpha and beta EEG power group differences-unimodal visual presentations.** Results for the alpha band **(left)** and beta band **(right)** are presented. Significant differences were determined using the Tukey–Kramer method for *p* < 0.05.

**FIGURE 6:**
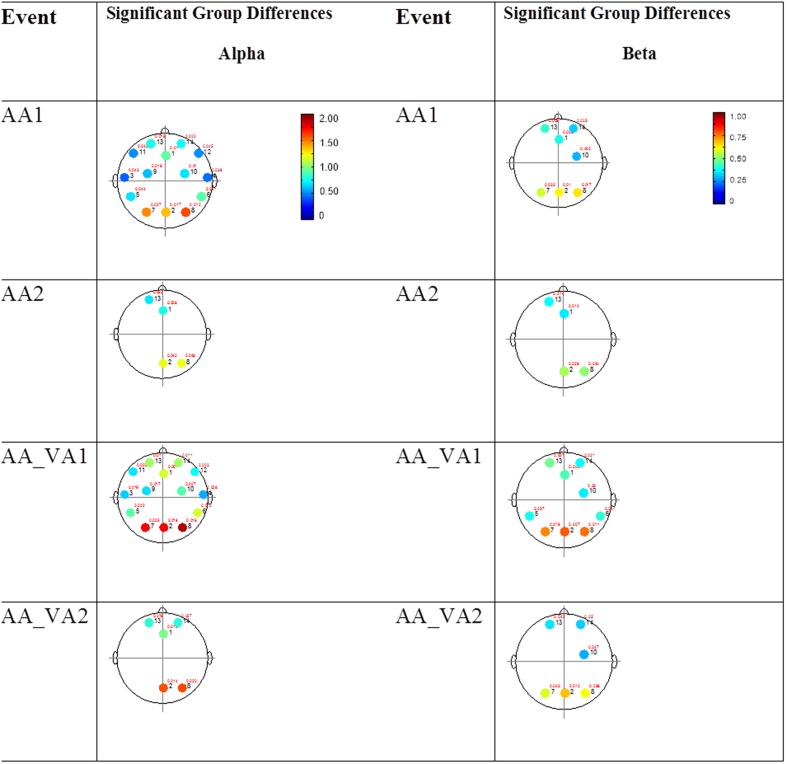
**Session 2 alpha and beta EEG power group differences-unimodal auditory presentations.** Results for the alpha band **(left)** and beta band **(right)** are presented. Significant differences were determined using the Tukey–Kramer method for *p* < 0.05.

**FIGURE 7:**
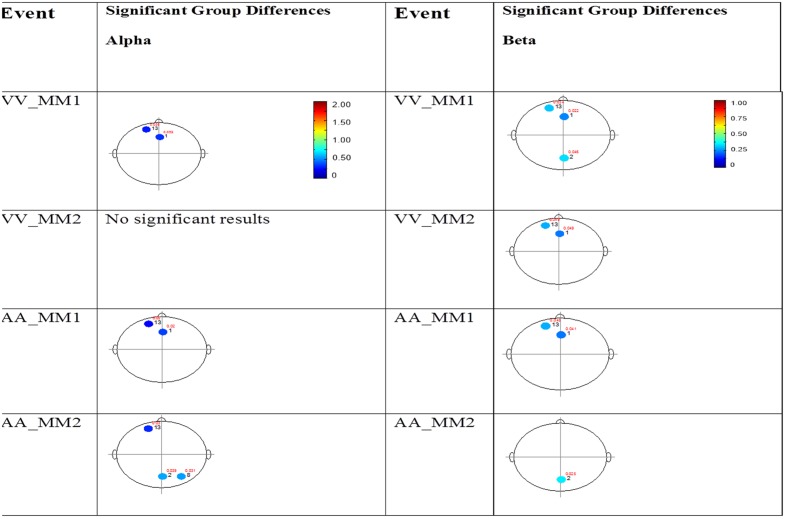
**Session 2 alpha and beta EEG power group differences-multimodal presentations.** Results for the alpha band **(left)** and beta band **(right)** are presented. Significant differences were determined using the Tukey–Kramer method for *p* < 0.05.

The results for the average power of the alpha and beta band frequencies for the ASD vs. NT groups are summarized in **Tables 4** and **5** as well as presented in **Figures 5–7**. **Figures 5** and **6** report the average power EEG head plots for alpha and beta bands in unimodal visual presentations and unimodal auditory presentations, respectively. **Figure 7** reports the average power for alpha and beta bands in mixed (simultaneous) presentation trials. Again, EEG average power was lower for the ASD group across all channels for all events (graphs of average EEG power). In this session, the unimodal presentations of auditory and visual stimuli (AA1, AA2, VV1, and VV2) were once again presented and were shown to elicit similar, although not entirely identical, EEG head plots compared to session 1. Plots of trials containing mixed (simultaneous) stimulus presentations were more similar to unimodal visual stimulus presentation and cross modal trials that were embedded in a visual block (VV_VA) than unimodal auditory or cross modal trials that were embedded in an auditory block (AA_VA). Differences in average power between the ASD and NT groups were consistently localized to reductions in both the alpha and beta bands in frontal areas (channel groups 1 and 13) for these trials; a profile which was consistent with the head plots observed during the presentation of unimodal visual stimuli in both sessions 1 and 2.

**Table 4:** Session 2 alpha EEG power event differences by group.

Comparison	Control		Autistic	
		
	Higher power	Region	Higher power	Region
AA1-AA_MM1	AA1	All		
AA1-AA_VA1	AA_VA1	1-FC, 2-PC, 3-LT, 4-RT, 5-LPA, 6-RPA, 7-LO, 10-RC, 12-RF, 13-LFP, 14-RFP		
AA2-AA_MM2	AA2	4-RT		
AA2-AA_VA2				
VV1-VV_MM1	VV1	2-PC		
VV1-VV_VA1				
VV2-VV_MM2	VV2	1-FC,13-LFP		
VV2-VV_VA2				


*1-FC, Frontal central; 2-PC, posterior central; 3-LT, left temporal area; 4-RT, right temporal; 5-LPA, left posterior auricular; 6-RPA, right posterior auricular; 7-LO, left occipital; 10-RC, right central; 12-RF, right frontal; 13-LFP, left frontal pole; 14-RFP, right frontal pole.*

**Table 5:** Session 2 beta EEG power event differences by group.

	Control		Autistic	
		
Comparison	Higher power	Region	Higher power	Region
AA1-AA_MM1	AA1	All		
AA1-AA_VA1	AA1_VA1	3-LT, 5-LPA, 7-LO, 12-RF		
AA2-AA_MM2	AA2	4-RT		
AA2-AA_VA2				
VV1-VV_MM1				
VV1-VV_VA1				
VV2-VV_MM2				
VV2-VV_VA2				


*3-LT, Left temporal; 4-RT, right temporal; 5-LPA, left posterior auricular; 7-LO, left occipital; 12-RF, right frontal.*

### BOLD fMRI

#### Single Stimulus Presentation Trials

**FIGURE 8:**
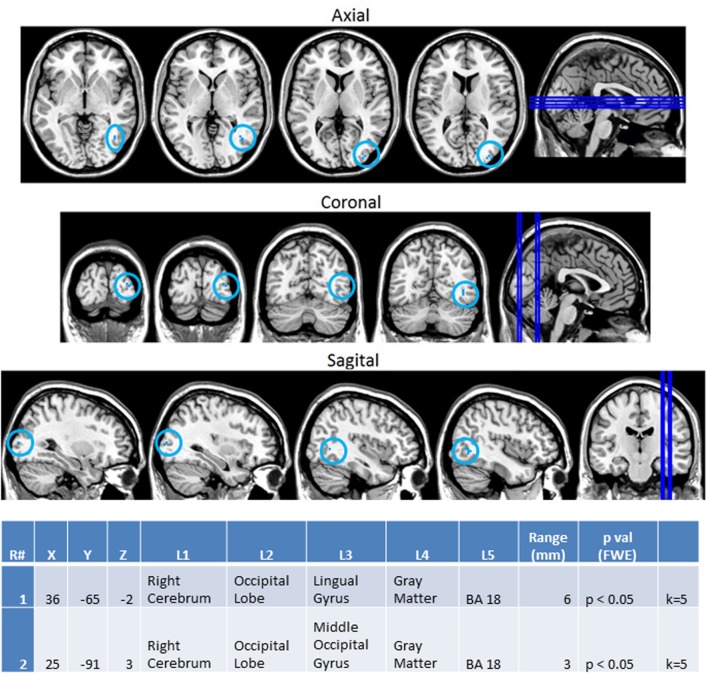
**Session 1 VV2-VA2 ASD > Controls significant fMRI activations.** Axial cross sections highlighting regions of greater ASD (blue) group-level activations are depicted for four planes. A sagittal cross section depicts the location of the axial planes (blue lines; **top row**). Coronal cross sections highlighting regions of greater ASD (blue) group-level activations are depicted for four planes. A sagittal cross section depicts the location of the coronal planes (blue lines; **middle row**). Sagittal cross sections highlighting regions of greater ASD (blue) group-level activations are depicted for four planes. A coronal cross section depicts the location of the sagittal planes (blue lines; **bottom row**).The corresponding Brodmann areas for significant areas of activation are listed in the table at bottom (under the heading “L5”). Statistical significance were based on a family wise error (FWE) corrected *p*-value threshold of *p* < 0.05 and k = 5 voxel threshold.

In order to better observe the effects of cross sensory unimodal processing compared to processing of a repeated presentation of a unimodal stimulus, we created contrast maps for the ASD and NT groups from the session 1 trials. From these contrasts we observed two conditions with significant fMRI activation differences between the NT and ASD groups: VV2-VA2 and AA2-VV2. **Figure 8** shows VV2-VA2 (a cross sensory unimodal processing condition) has significant fMRI activations for ASD>NT group. This contrast examined differences in the second visual stimulus in a sequence of two visual stimuli and the audio stimuli in a visual audio sequence. The ASD group exhibited greater activation of the right sided lingual and middle occipital gyri (Brodmann area 18; BA 18R). This is of particular interest as it represents the contrast in activity from an auditory presentation (VA2) that followed a visual presentation vs. a visual presentation that followed a visual presentation (VV2), thereby highlighting the greater recruitment of occipital structures in ASD vs. NT despite the change from a visual to auditory stimulus.

**FIGURE 9:**
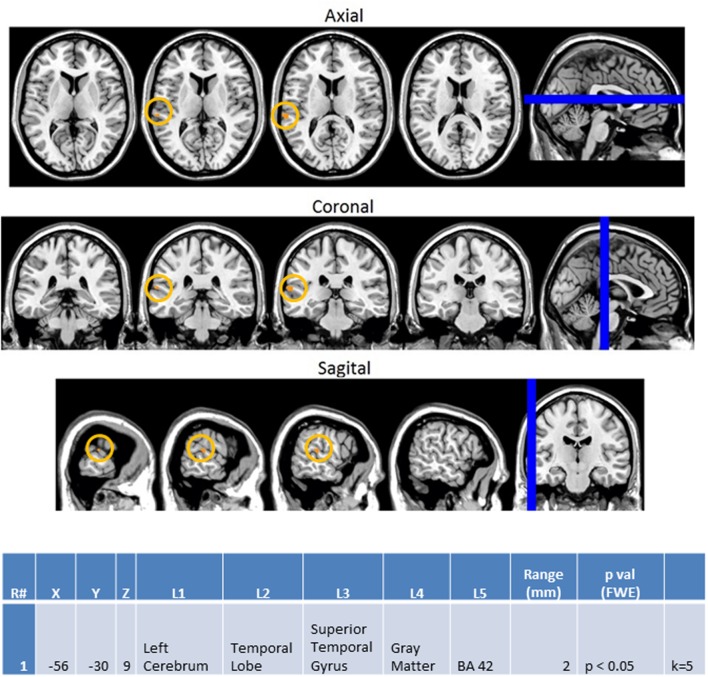
**Session 1 AA2-VV2 Controls > ASD significant fMRI activations.** Axial cross sections highlighting regions of greater Control (orange) group-level activations are depicted for four planes. A sagittal cross section depicts the location of the axial planes (blue lines; **top row**). Coronal cross sections highlighting regions of greater Control (orange) group-level activations are depicted for four planes. A sagittal cross section depicts the location of the coronal planes (blue lines; **middle row**). Sagittal cross sections highlighting regions of greater Control (orange) group-level activations are depicted for four planes. A coronal cross section depicts the location of the sagittal planes (blue lines; **bottom row**). The corresponding Brodmann area for significant areas of activation is listed in the table at bottom (under the heading “L5”). Statistical significance were based on a FWE corrected *p*-value threshold of *p* < 0.05 and k = 5 voxel threshold.

Next, we investigated how processing of repeated presentation of a unimodal stimulus may be affected by contrasting unimodal processing of auditory and visual stimuli trial conditions across the ASD and NT groups in **Figure 9**. In this contrast, the activations related to the second auditory stimulus in a sequence of two auditory stimuli were subtracted from the second visual stimulus in a sequence of two visual stimuli. Here we see that controls exhibited higher activation in unimodal processing of auditory information (AA2-VV2) with predictable increased activation of the left auditory cortex (BA 42L).

#### Mixed (Simultaneous) Presentation Trials

**FIGURE 10:**
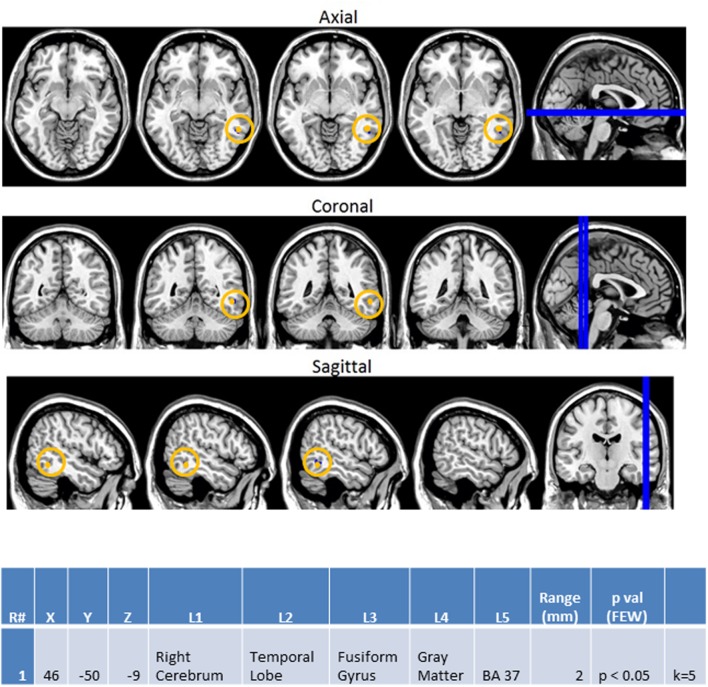
**Session 2 AA_MM2-AA2 Control > ASD significant fMRI activations.** Axial cross sections highlighting regions of greater Control (orange) group-level activations are depicted for four planes. A sagittal cross section depicts the location of the axial planes (blue lines; **top row**). Coronal cross sections highlighting regions of greater Control (orange) group-level activations are depicted for four planes. A sagittal cross section depicts the location of the coronal planes (blue lines; **middle row**). Sagittal cross sections highlighting regions of greater Control (orange) group-level activations are depicted for four planes. A coronal cross section depicts the location of the sagittal planes (blue lines; **bottom row**). The corresponding Brodmann area for significant area of activation is listed in the table at bottom (under the heading “L5”). Statistical significance were based on a FWE corrected *p*-value threshold of *p* < 0.05 and k = 5 voxel threshold.

Finally, the same methodology was used to create contrast maps to observe the effects of multimodal stimulus presentation during session 2. From this, AA_MM2-AA2 was the only contrast map to show significance. As seen in **Figure 10**, it showed greater activity in the NT than ASD group for the right fusiform gyrus (BA 37R). This contrast consists of activation during the second presentation of a sequence of two multimodal stimuli minus the second presentation in a sequence of two audio stimuli. In this case the multimodal stimuli were surprise stimuli that were applied to only a small percentage of a larger stimulus block made of mostly sequences of two audio stimuli followed by another sequence of two audio stimuli.

#### EEG Beta Power Correlations to fMRI Findings

**Supplementary Figure S3** illustrates the EEG beta power plots for the relevant channels corresponding to the regions of interest observed in the fMRI results. EEG beta power plots are consistent with the fMRI observations for both session 1 contrasts (VA2-VV2 and AA2-VV2). However, the EEG beta power plot for the session 2 contrast AA_MM2-AA2 shows higher beta power in the temporal region amongst the ASD group; a finding which is inconsistent with the fMRI results.

## Discussion

### Interpretation of Behavioral Observations

Behaviorally, we see that adults with ASD were shown to perform equally as accurately across all trial types. The lack of a consistent difference across trial types led us to conclude that similar to the works of van der Smagt et al. (2007) and Mongillo et al. (2008) the use of pure tones and flashes was successfully simple enough to elicit similar behavioral responses between both groups; allowing us to better assess differences in how their brains processed the stimuli without major reservations concerning the adults with ASD’s lack of higher-level comprehension of the task at hand affecting our interpretation.

### EEG Observations Suggest Greater Deficits in Auditory Processing Relative to Visual Processing in Adults with ASD

From our EEG recordings we consistently observed that trials containing an auditory component displayed lower average power across both the alpha and beta frequencies in nearly all channel groupings (frontal, temporal, and occipital). The indirect incorporation of a visual stimulus, as seen in the cross modal trials (VA2 and AA_VA2), led to fewer areas of under-activation and the use of simultaneous presentation of audiovisual stimuli (AA_MM1, AA_MM2) resulted in near total compensation. Furthermore, simultaneous audiovisual presentation closely resembled unimodal visual stimulus presentation, suggesting that processing of visual flashes by adults with ASD was far more similar to NTs than the processing of auditory tones. Although, deficits in both auditory and visual domains have been previously reported, to our knowledge this is the first study to explicitly demonstrate the individual deficits of these modalities within the same experimental paradigm. Within this paradigm we see that the incorporation of visual flashes allowed this group of adults with ASD to process stimuli in a manner similar to NTs.

### BOLD fMRI Supports EEG Observations of Greater Disparity during Unimodal vs. Multimodal Presentation

Blood oxygen level dependent functional magnetic resonance imagining observations in this study focused on contrasting differences between the ASD and NT groups in terms of how processing differed between the presentation types. In accordance with our EEG observations discussed above, the heightened left auditory cortex activation (BA 42L) observed in the NT group during the unimodal contrast of AA2-VV2 aligns with previous research which associated BA 42L with auditory tone processing (Menéndez-Colino et al., 2007) and corroborates our behavioral and EEG power observations of the ASD group being less accurate and showing reduced power in response to auditory rather than visual stimuli.

Furthermore, the ASD group exhibited increased activation in the right lingual and middle occipital gyri (BA 18R) during VV2-VA2; a finding that was also consistent with our EEG findings (**Supplementary Figure S3**). This condition represents cross sensory unimodal processing and these areas have been previously associated with visual pattern matching (Pessoa et al., 2002) and non-conscious visual processing (Slotnick and Schacter, 2006). Such findings suggest that the ASD group persisted in engaging visual processing centers during the presentation of auditory stimuli. This suggests a greater reliance on visual information, in congruence with our other observations, as well as a difficulty in switching between visual and auditory information.

Finally, evidence from our BOLD fMRI contrasts of the unimodal and mixed (simultaneous) stimulus presentations demonstrated the full effect of this difference in visual and auditory processing. Given that the AA_MM2-AA2 represented a situation in which the second presentation of simultaneous audiovisual stimuli were hidden inside of an all auditory block and were contrasted to the second presentation of auditory stimuli within a unimodal auditory block this condition allowed for the assessment of visual aid within an auditory context. Although, our EEG data showed that incorporation of visual stimuli was associated with reduced differences in power between the NT and ASD groups (in fact, the ASD group showed higher EEG beta power than the NTs; **Supplementary Figure S3**) NTs were shown to have increased activation of the right fusiform gyrus (BA 37R) during this contrast. This area has been associated with selective attention (Le et al., 1998) and visual recognition of objects (Slotnick and Schacter, 2006; in this case, presumably, the oval shape of the flash). Its activity in NTs suggests they are maintaining attention on the visual aspect of patterns and utilizing visual memory more than their ASD counterparts. It is therefore possible that while the incorporation of visual stimuli lessens the difference between the two groups NTs benefit more so than ASDs. This may in turn explain the paradoxical heightened EEG beta power observed in ASDs for this condition. The heightened EEG beta power may represent a higher level of diffuse temporal lobe activity that is necessary to compensate for the lack of activity in the right fusiform gyrus.

The current study attempted to assess non-human multimodal auditory and visual stimulus processing in adults with ASD through the use of EEG and BOLD fMRI. Recognizing that this would be a multi-level process, stimuli were assessed in unimodal, cross-modal, and multimodal presentations. In an effort to further dissect the potential differences in sensory processing between ASD and NT groups, the mode of stimulus presentation, as well as the order in which it was presented (“first” or “second”) was examined. Using this methodical approach to stimulus presentation allowed for better interpretation of how each level of processing (i.e., assessment of simple sensory processing via unimodal stimulus presentation and assessment of lower-level sensory integration via presentation of cross modal and multimodal stimuli) was affected in these individuals with the added confidence that these observations were made within the same individuals within the same experimental parameters using two forms of neuroimaging assessment.

Surprisingly, increasing levels of complexity (i.e., unimodal→cross modal→multimodal) in stimulus presentation did not result in heightened deficits in ASD performance. Instead, through the use of this multilevel paradigm, we observed the greatest sources of difference in processing originated from unimodal processing; with auditory stimuli producing greater deficits than visual stimuli. Here it may be seen that although each level of stimulus presentation had unique processing deficits in the ASD group, these differences were minimized, *not maximized*, through the use of simultaneous audiovisual presentation. These findings may offer additional understanding to the reported success of the integration of music into teaching and therapy for individuals with ASD as simultaneous presentation of visual cues with auditory signals may lead to more successful processing for these individuals. (refer to Wan et al., 2010 for review). When contrasting this finding to previous studies that suggested simultaneous presentation of audiovisual stimuli led to greater deficits, it is likely that such stimuli must be self-congruent (such as the multimodal stimuli used herein as well as music in general) to assist those with ASD and that conflicting stimuli (where the visual and audio stimuli directly contrast each other) may exacerbate these deficiencies. A separate investigation of the effects of conflicting vs. compatible simultaneous audiovisual presentation will be necessary to address this issue.

### Limitations

The current study represents an attempt to identify neural correlates of sensory processing through the use of pure tones and flashes in adults with ASD compared to NT controls using EEG and BOLD fMRI. Although, clear differences in neural correlates were observed between the ASD and NT groups, it is important to remember that autism is a spectrum representing a very diverse series of symptoms with varying severity. As such, limitations of this study include that we were not privy to documentation of the symptoms nor their severity displayed by our participants, it only investigated adults, did not consider genotyping for known associated alleles, or use other biomarkers that may further play a role in classification of participants for neuroimaging studies (Lenroot and Yeung, 2013). It is important to note that while we found significant observations for simultaneous presentation of auditory and visual stimuli contrasted to other modes of presentation the small number of mixed trials per participant (3) causes us to proceed with caution in our generalization of these findings. Even though blocks of stimulus presentation were counterbalanced across subjects and each block consisted of 20 trials, the lack of internal repetition of a given block of stimuli for a given individual is reason as well to limit the scope of our interpretation. Likewise, the uneven sex distribution between our NT and ASD groups represents a potential confound. Furthermore, trials in which no response was made were discarded, thereby potentially skewing the reported behavioral results. This was, however, necessary as including these trials would undoubtedly skew the neuroimaging results leading to inaccurate assessment of sensory processing. Additionally, we recognize that an ISI of 1.3 s may be too short to allow for optimal decomposition of activation signal from one trial to another. We therefore recommend use of a longer ISI for related future endeavors. Finally, previous studies have shown that individuals with ASD are known to display behavior and physiology that is more similar to NTs when the stimuli are devoid of human social cues (Mongillo et al., 2008); as was the case in this investigation. Therefore, the degree to which simultaneous multimodal presentation is beneficial may be limited to non-human stimuli.

### Directions for Future Work

Replication of the findings observed in this study with a stricter inclusion/exclusion criteria that subcategorized groups on known genotypic markers or standardized psychological test scores and comparison of human and non-human stimuli is warranted before broader generalization can be applied to individuals with ASD. Further attempts to contrast the performance of individuals with ASD to those with other forms of developmental delay or social impairment may lead to a more accurate understanding of how sensory processing is functionally impaired in individuals of these respective groups. Additionally, analyzing the trial conditions by pattern type (e.g., constant, increasing, decreasing, or crescendo-decrescendo) may give insight into differential processing of stimuli across mode of presentation (e.g., visual, auditory, or audiovisual). Finally, the use of Diffusion Tensor Imaging and ICA across cross sectional age groups may allow for assessment of functional connectivity of sensory processing, thereby creating a fuller picture of how processing is affected by structural and functional neurobiological underpinnings.

## Conclusion

Our observations led us to conclude two major points. First, greater differences in sensory processing between ASD and NT groups existed for presentation of auditory rather than visual stimuli, as evidenced by our EEG and BOLD fMRI findings. Second, the presentation of multimodal stimuli minimized the differences seen between the ASD and NT groups; as evidenced by EEG and BOLD fMRI. This investigation has led to a greater understanding of how adults with ASD process non-human audiovisual stimuli and suggests that simultaneous presentation of such stimuli may result in the most similar sensory processing experience compared to NTs.

## Author Contributions

MB, MO’B, and DR designed the experiment. MO’B provided a framework for the experimental protocol and stimuli and provided expertise on fMRI acquisition, analysis, and results. SZ and BM conducted sound calibration and testing, conducted auditory tests, and provided expertise on human auditory response. DR provided expertise on autism and sensory integration. EH, BM, and RCA, and MB conducted subject recruitment, acquired the EEG and fMRI data, and ran the initial fMRI and EEG analyses. MB wrote the initial draft of the paper. RR provided interpretation and analysis of the data and results and wrote the complete final draft of the paper, including all of the discussion and literature review sections. DR, RCA, and MB provided edits for and review of the final draft of the paper. MB and RR provided final revisions.

## Conflict of Interest Statement

The authors declare that the research was conducted in the absence of any commercial or financial relationships that could be construed as a potential conflict of interest.

## Supplementary Material

**FIGURE S1 | Task accuracy for EEG sessions.** EEG Sensory Task session 1 **(Top)** and session 2 **(Bottom)** accuracy scores. SVV, “same” visual–visual, DVV, “different” visual–visual, SAA, “same” audio–audio, DAA, “different” audio–audio, SVA, “same” visual–audio, DVA, “different” visual–audio. No significant group differences were observed.

**FIGURE S2 | Task accuracy for fMRI sessions.** fMRI Sensory Task session 1 **(Top)** and session 2 **(Bottom)** accuracy scores. SVV, “same” visual–visual, DVV, “different” visual–visual, SAA, “same” audio–audio, DAA, “different” audio–audio, SVA, “same” visual–audio, DVA, “different” visual–audio. No significant group differences were observed.

**FIGURE S3 | EEG beta Power plots corresponding to fMRI contrast conditions of significant activation.** EEG Power plots are depicted for the beta band frequencies across the relevant channels respective to the regions of interests identified for the fMRI contrasts: VV2-VA2 **(Top)**, AA2-VV2 **(Middle)**, and AA_MM_2-AA2 **(Bottom)** for both the ASD and NT groups.

## References

[B1] Amaral D. G. Schumann C. M. Nordahl C. W. (2008). Neuroanatomy of autism. *Trends Neurosci.* 31 137–145. 10.1016/j.tins.2007.12.005 18258309

[B2] American Psychiatric Association. (2013). *Diagnostic and Statistical Manual of Mental Disorders: DSM-V.* 5th Edn Washington, DC: American Psychiatric Association.

[B3] Anagnostou E. Taylor M. J. (2011). Review of neuroimaging in autism spectrum disorders: what have we learned and where we go from here. *Mol. Autism* 2:4 10.1186/2040-2392-2-4 PMC3102613 21501488

[B4] Annaz D. Remington A. Milne E. Coleman M. Campbell R. Thomas M. S. (2010). Development of motion processing in children with autism. *Dev. Sci.* 13 826–838. 10.1111/j.1467-7687.2009.00939.x 20977554

[B5] Asperger H. (1944). [Autistic psychopathy in childhood]. *Arch Psychiatr Nervenkr* 117 76–136. 10.1007/BF01837709

[B6] Baranek G. T. David F. J. Poe M. D. Stone W. L. Watson L. R. (2006). Sensory experiences questionnaire: discriminating sensory features in young children with autism, developmental delays, and typical development. *J. Child Psychol. Psychiatry* 47 591–601. 10.1111/j.1469-7610.2005.01546.x 16712636

[B7] Ben-Sasson A. Hen L. Fluss R. Cermak S. A. Engel-Yeger B. Gal E. (2009). A meta-analysis of sensory modulation symptoms in individuals with autism spectrum disorders. *J. Autism. Dev. Disord.* 39 1–11. 10.1007/s10803-008-0593-3 18512135

[B8] Blakemore S. J. Tavassoli T. Calo S. Thomas R. M. Catmur C. Frith U. (2006). Tactile sensitivity in Asperger syndrome. *Brain Cogn.* 61 5–13. 10.1016/j.bandc.2005.12.013 16500009

[B9] Courchesne E. Lincoln A. J. Kilman B. A. Galambos R. (1985). Event-related brain potential correlates of the processing of novel visual and auditory information in autism. *J. Autism. Dev. Disord.* 15 55–76. 10.1007/BF01837899 3980430

[B10] Crane L. Goddard L. Pring L. (2009). Sensory processing in adults with autism spectrum disorders. *Autism* 13 215–228. 10.1177/1362361309103794 19369385

[B11] Dalton K. M. Nacewicz B. M. Alexander A. L. Davidson R. J. (2007). Gaze-fixation, brain activation, and amygdala volume in unaffected siblings of individuals with autism. *Biol. Psychiatry* 61 512–520. 10.1016/j.biopsych.2006.05.019 17069771

[B12] Dalton K. M. Nacewicz B. M. Johnstone T. Schaefer H. S. Gernsbacher M. A. Goldsmith H. H. (2005). Gaze fixation and the neural circuitry of face processing in autism. *Nat. Neurosci.* 8 519–526. 15750588 10.1038/nn1421 PMC4337787

[B13] De Jonge M. V. Kemner C. De Haan E. H. Coppens J. E. Van den Berg T. J. T. P. Van Engeland H. (2007). Visual information processing in high-functioning individuals with autism spectrum disorders and their parents. *Neuropsychology* 21 65–73. 10.1037/0894-4105.21.1.65 17201530

[B14] Dunn M. A. Gomes H. Gravel J. (2008). Mismatch negativity in children with autism and typical development. *J. Autism. Dev. Disord.* 38 52–71. 10.1007/s10803-007-0359-3 17624605

[B15] Foss-Feig J. H. Kwakye L. D. Cascio C. J. Burnette C. P. Kadivar H. Stone W. L. (2010). An extended multisensory temporal binding window in autism spectrum disorders. *Exp. Brain Res.* 203 381–389. 10.1007/s00221-010-2240-4 20390256 PMC2871100

[B16] Foxe J. J. Molholm S. (2009). Ten years at the multisensory forum: musings on the evolution of a field. *Brain Topogr.* 21 149–154. 10.1007/s10548-009-0102-9 19452270

[B17] Iarocci G. McDonald J. (2006). Sensory integration and the perceptual experience of persons with autism. *J. Autism. Dev. Disord.* 36 77–90. 10.1007/s10803-005-0044-3 16395537

[B18] Iarocci G. Rombough A. Yager J. Weeks D. J. Chua R. (2010). Visual influences on speech perception in children with autism. *Autism* 14 305–320. 10.1177/1362361309353615 20591957

[B19] Jemel B. Mimeault D. Saint-Amour D. Hosein A. Mottron L. (2010). VEP contrast sensitivity responses reveal reduced functional segregation of mid and high filters of visual channels in autism. *J. Vis.* 10 13 10.1167/10.6.13 20884562

[B20] Källstrand J. Olsson O. Nehlstedt S. F. Sköld M. L. Nielzén S. (2010). Abnormal auditory forward masking pattern in the brainstem response of individuals with Asperger syndrome. *Neuropsychiatr. Dis. Treat.* 6 289–296. 20628629 10.2147/ndt.s10593 PMC2898167

[B21] Kana R. K. Wadsworth H. M. Travers B. G. (2011). A systems level analysis of the mirror neuron hypothesis and imitation impairments in autism spectrum disorders. *Neurosci. Biobehav. Rev.* 35 894–902. 10.1016/j.neubiorev.2010.10.007 20974171

[B22] Kanner L. (1943). Autistic disturbances of affective contact. *Nerv. Child* 2 217–250. 4880460

[B23] Koh H. C. Milne E. Dobkins K. (2010). Spatial contrast sensitivity in adolescents with autism spectrum disorders. *J. Autism. Dev. Disord.* 40 978–987. 10.1007/s10803-010-0953-7 20213250

[B24] Kwon S. Kim J. Choe B. H. Ko C. Park S. (2007). Electrophysiologic assessment of central auditory processing by auditory brainstem responses in children with autism spectrum disorders. *J. Korean Med. Sci.* 22 656–659. 10.3346/jkms.2007.22.4.656 17728505 PMC2693815

[B25] Le T. H. Pardo J. V. Hu X. (1998). 4 T-fMRI study of nonspatial shifting of selective attention: cerebellar and parietal contributions. *J. Neurophysiol.* 79 1535–1548. 9497430 10.1152/jn.1998.79.3.1535

[B26] Leekam S. R. Nieto C. Libby S. J. Wing L. Gould J. (2007). Describing the sensory abnormalities of children and adults with autism. *J. Autism. Dev. Disord.* 37 894–910. 10.1007/s10803-006-0218-7 17016677

[B27] Lenroot R. K. Yeung P. K. (2013). Heterogeneity within autism spectrum disorders: what have we learned from neuroimaging studies? *Front. Hum. Neurosci.* 7:733 10.3389/fnhum.2013.00733 PMC3812662 24198778

[B28] Marco E. J. Hinkley L. B. Hill S. S. Nagarajan S. S. (2011). Sensory processing in autism: a review of neurophysiologic findings. *Pediatr. Res.* 69 48R–54R. 10.1203/PDR.0b013e3182130c54 PMC3086654 21289533

[B29] Menéndez-Colino L. M. Falcón C. Traserra J. Berenguer J. Pujol T. Doménech J. (2007). Activation patterns of the primary auditory cortex in normal-hearing subjects: a functional magnetic resonance imaging study. *Acta Otolaryngol.* 127 1283–1291. 10.1080/00016480701258705 17851933

[B30] Minshew N. J. Sweeney J. Luna B. (2002). Autism as a selective disorder of complex information processing and underdevelopment of neocortical systems. *Mol. Psychiatry* 7 S14–S15. 10.1038/sj.mp.4001166 12142935

[B31] Mongillo E. A. Irwin J. R. Whalen D. H. Klaiman C. Carter A. S. Schultz R. T. (2008). Audiovisual processing in children with and without autism spectrum disorders. *J. Autism. Dev. Disord.* 38 1349–1358. 10.1007/s10803-007-0521-y 18307027

[B32] Oldfield R. C. (1971). The assessment and analysis of handedness: the Edinburgh inventory. *Neuropsychologia* 9 97–113. 10.1016/0028-3932(71)90067-4 5146491

[B33] Parron C. Da Fonseca D. Santos A. Moore D. Monfardini E. Deruelle C. (2008). Recognition of biological motion in children with autistic spectrum disorders. *Autism* 12 261–274. 10.1177/1362361307089520 18445735

[B34] Pessoa L. Gutierrez E. Bandettini P. A. Ungerleider L. G. (2002). Neural correlates of visual working memory: fMRI amplitude predicts task performance. *Neuron* 35 975–987. 10.1016/S0896-6273(02)00817-6 12372290

[B35] Rosenhall U. Nordin V. Brantberg K. Gillberg C. (2003). Autism and auditory brain stem responses. *Ear Hear.* 24 206–214. 10.1097/01.AUD.0000069326.11466.7E 12799542

[B36] Russo N. Nicol T. Trommer B. Zecker S. Kraus N. (2009). Brainstem transcription of speech is disrupted in children with autism spectrum disorders. *Dev. Sci.* 12 557–567. 10.1111/j.1467-7687.2008.00790.x 19635083 PMC2718770

[B37] Russo N. M. Skoe E. Trommer B. Nicol T. Zecker S. Bradlow A. (2008). Deficient brainstem encoding of pitch in children with autism spectrum disorders. *Clin. Neurophysiol.* 119 1720–1731. 10.1016/j.clinph.2008.01.108 18558508 PMC2536645

[B38] Sanchez-Marin F. J. Padilla-Medina J. A. (2008). A psychophysical test of the visual pathway of children with autism. *J. Autism. Dev. Disord.* 38 1270–1277. 10.1007/s10803-007-0507-9 18058009

[B39] Schultz R. T. (2005). Developmental deficits in social perception in autism: the role of the amygdala and fusiform face area. *Int. J. Dev. Neurosci.* 23 125–141. 10.1016/j.ijdevneu.2004.12.012 15749240

[B40] Slotnick S. D. Schacter D. L. (2006). The nature of memory related activity in early visual areas. *Neuropsychologia* 44 2874–2886. 10.1016/j.neuropsychologia.2006.06.021 16901520

[B41] Smith E. G. Bennetto L. (2007). Audiovisual speech integration and lipreading in autism. *J. Child Psychol. Psychiatry* 48 813–821. 10.1111/j.1469-7610.2007.01766.x 17683453

[B42] Tomchek S. D. Dunn W. (2007). Sensory processing in children with and without autism: a comparative study using the short sensory profile. *Am. J. Occup. Ther.* 61 190–200. 10.5014/ajot.61.2.190 17436841

[B43] van der Smagt M. J. van Engeland H. Kemner C. (2007). Brief report: can you see what is not there? Low-level auditory–visual integration in autism spectrum disorder. *J. Autism. Dev. Disord.* 37 2014–2019. 17273934 10.1007/s10803-006-0346-0

[B44] Vandenbroucke M. W. Scholte H. S. van Engeland H. Lamme V. A. Kemner C. (2008). A neural substrate for atypical low-level visual processing in autism spectrum disorder. *Brain* 131 1013–1024. 10.1093/brain/awm321 18192288

[B45] Wan C. Y. Demaine K. Zipse L. Norton A. Schlaug G. (2010). From music making to speaking: engaging the mirror neuron system in autism. *Brain Res. Bull.* 82 161–168. 10.1016/j.brainresbull.2010.04.010 20433906 PMC2996136

