# Supplementary figures and images for: Visual, Auditory, and Cross Modal Sensory Processing in Adults with Autism: An EEG Power and BOLD fMRI Investigation
Source: Front Hum Neurosci. 2016 Apr 19;10:167. doi: 10.3389/fnhum.2016.00167 (PMC4835455; doi:10.3389/fnhum.2016.00167)

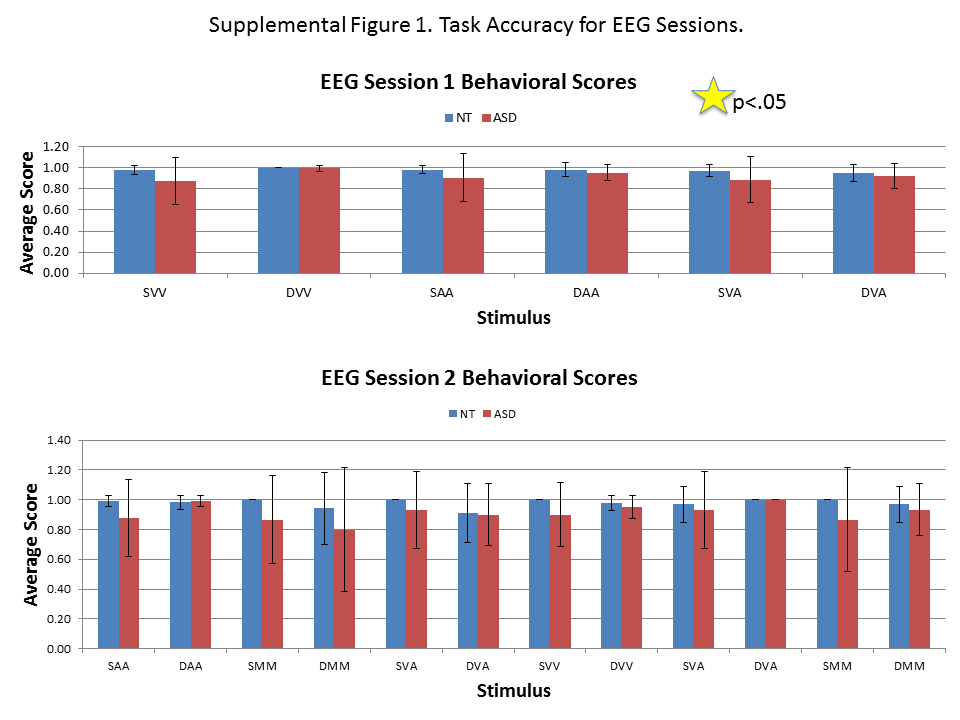

Supplement: Supplementary file 1 [file Image_1.TIF]

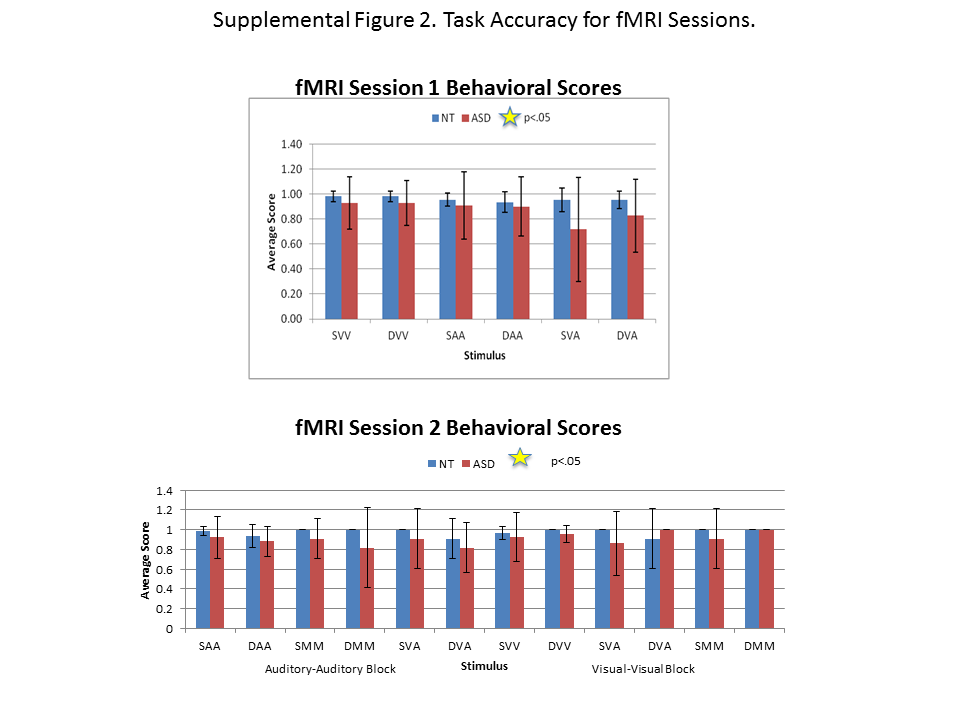

Supplement: Supplementary file 2 [file Image_2.TIF]

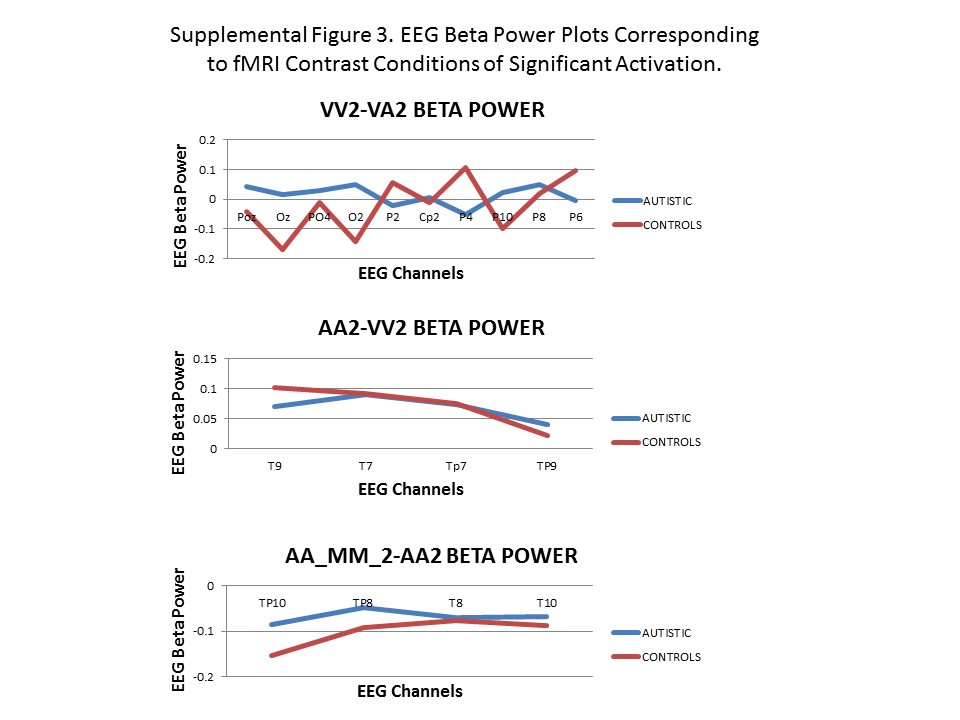

Supplement: Supplementary file 3 [file Image_3.TIF]
